# Entrectinib dose confirmation in pediatric oncology patients: pharmacokinetic considerations

**DOI:** 10.1007/s00280-023-04510-1

**Published:** 2023-03-08

**Authors:** Georgina Meneses-Lorente, Elena Guerini, Francois Mercier, Neil Parrott, Karey Kowalski, Edna Chow-Maneval, Vincent Buchheit, Guillaume Bergthold, Elizabeth Fox, Alex Phipps, Nassim Djebli

**Affiliations:** 1 Pharma Research and Early Development, Roche Products Ltd., Welwyn Garden City, UK; 2 Pharma Research and Early Development, Roche Innovation Center Basel, F. Hoffmann-La Roche Ltd., Basel, Switzerland; 3 Biostatistics, Roche Innovation Center Basel, F. Hoffmann-La Roche Ltd., Basel, Switzerland; 4 PharmD Clinical Pharmacology, Ignyta, Inc., San Diego, CA USA; 5 Clinical Development, Ignyta, Inc., San Diego, CA USA; 6 Product Development Oncology and Hematology Department, F. Hoffmann-La Roche Ltd., Basel, Switzerland; 7 Department of Oncology, St Jude Children’s Research Hospital, Memphis, TN USA; 8 Department of Clinical Pharmacology, Roche Innovation Center Welwyn, Roche Products Ltd., Welwyn Garden City, UK

**Keywords:** Entrectinib, TRK/ROS1/ALK, Pharmacokinetics, Pediatrics

## Abstract

**Purpose:**

Entrectinib is a central nervous system-active potent inhibitor of tropomyosin receptor kinase (TRK), with anti-tumor activity against neurotrophic *NTRK* gene fusion-positive tumors. This study investigates the pharmacokinetics of entrectinib and its active metabolite (M5) in pediatric patients and aims to understand whether the pediatric dose of 300 mg/m^2^ once daily (QD) provides an exposure that is consistent with the approved adult dose (600 mg QD).

**Methods:**

Forty-three patients aged from birth to 22 years were administered entrectinib (250–750 mg/m^2^ QD) orally with food in 4-week cycles. Entrectinib formulations included capsules without acidulant (F1) and capsules with acidulant (F2B and F06).

**Results:**

Although there was interpatient variability with F1, entrectinib and M5 exposures increased dose dependently. Lower systemic exposures were observed in pediatric patients receiving 400 mg/m^2^ QD entrectinib (F1) versus adults receiving either the same dose/formulation or the recommended flat dose of 600 mg QD (~ 300 mg/m^2^ for a 70 kg adult) due to suboptimal F1 performance in the pediatric study. The observed pediatric exposures following 300 mg/m^2^ QD entrectinib (F06) were comparable to those in adults receiving 600 mg QD.

**Conclusions:**

Overall, the F1 formulation of entrectinib was associated with lower systemic exposure in pediatric patients compared with the commercial acidulant formulation (F06). Systemic exposures achieved in pediatric patients with the F06 recommended dose (300 mg/m^2^) were within the known efficacious range in adults, confirming the adequacy of the recommended dose regimen with the commercial formulation.

## Introduction

Entrectinib is a potent, central nervous system (CNS)-active inhibitor of tyrosine receptor kinases (TRK) A, B and C, ROS proto-oncogene 1 receptor tyrosine kinase, and anaplastic lymphoma kinase (ALK) [1–3]. Entrectinib has been approved for the treatment of adult and pediatric (> 12 years) patients with tumors that harbor neurotrophic TRK (*NTRK*) 1/2/3 gene fusions and adults with *ROS1*-positive non-small cell lung cancer (NSCLC) [4–6]. Studies with entrectinib in adult patients included the supporting and pivotal studies STARTRK-1 (NCT02097810) and STARTRK-2 (NCT02568267), respectively. Study STARTRK-NG (NCT02650401) is an ongoing study in pediatric patients.

Entrectinib is a basic, lipophilic molecule with significant pH-dependent solubility. A previous pharmacokinetic study has demonstrated that entrectinib is the major circulating entity and M5 (N-demethylated) is a major active (equipotent) circulating metabolite [7]. The metabolism of entrectinib is equivalent in adult and pediatric patients [7]. Initial studies, including pivotal and supporting studies, with entrectinib employed a gelatin capsule formulation (F1). The F1 formulation did not contain an acidulant, which made this formulation very sensitive to gastric conditions such as concomitant food intake, and increased pH caused by comedications. As a result, the performance of the F1 formulation was sub-optimal during clinical dosing in the adult patient population due to high variability in systemic exposure [7, 8]. The F1 formulation was therefore not considered appropriate for commercial formulation and an alternative gelatin capsule formulation (F2A) was developed. The F2A formulation contained an acidulant (betaine hydrochloride) in order to reduce the sensitivity and variability of entrectinib bioavailability to gastric pH conditions. The F2A formulation was used in the final stage of the adult study STARTRK-1 and in the pivotal adult study STARTRK-2 as well as in early supporting clinical pharmacology studies [7, 9]. Consequently, during development, the majority of adult patients received the F2A capsules as the primary clinical formulation. Subsequently, a capsule formulation (F06) containing an alternative acidulant (tartaric acid) was developed as the commercial formulation to improve global regulatory acceptability. The F2A and F06 formulations have been shown to be bioequivalent [7].

The F06 formulation was used in later supporting clinical pharmacology studies, but was not used in clinical safety/efficacy studies before the clinical cutoff (May 31, 2018) for the global submissions; therefore, all the available data during development in the adult patient population was with F2A. Addition of acidulants effectively resolved the variability issues observed with F1, and neither F2A nor F06 are influenced by changes in gastric conditions.

The F2A formulation was only available as a 200 mg dose strength, whereas F1 was available at 100–200 mg dose strengths. Therefore, the ongoing pediatric study (STARTRK-NG) initially used the F1 formulation due to the availability of a 100 mg dose strength needed to dose pediatric patients. In addition, the lack of acidulant in F1 capsules made them feasible to be opened and sprinkled into food for those pediatric patients unable to swallow a capsule. For the lowest dose level (250 mg/m^2^), another formulation (F2B) was used, which was similar to F2A (i.e., it included an acidulant and could not be opened), but was also available as a 100 mg dose strength. F06 was subsequently introduced into the pediatric study in June 2019 for patients who were able to swallow capsules.

The purpose of this article is to describe the noncompartmental pharmacokinetics of entrectinib administered primarily as the F1 formulation in pediatric patients; to graphically compare the systemic exposure of entrectinib in pediatric patients with adult patients to contextualize the differences between the populations when receiving F1; and to graphically compare the systemic exposure of entrectinib administered as the F1 and F06 formulations in pediatric patients who responded to treatment according to RECIST criteria to further confirm the adequacy of the recommended dose regimen with the commercial formulation (F06). Given the nature of the study and the small sample sizes, it should be noted that no statistical comparisons were conducted.

## Materials and methods

### Study design

Study STARTRK-NG is an ongoing open-label, dose escalation and expansion study of entrectinib in children and adolescents with relapsed or refractory extracranial solid tumors or primary CNS tumors with TRK or ROS1 gene fusions. The dose escalation part allowed inclusion of patients without gene fusions. The study design is summarized here; further details and the safety and efficacy results have been reported separately [10]. During dose escalation, patients received entrectinib orally with food, once daily (QD) in repeated 4-week cycles. Four dose levels (250, 400, 550 and 750 mg/m^2^) of entrectinib were investigated as per a standard 3 + 3 patient enrollment scheme. The lowest dose (250 mg/m^2^) was administered as the F2B capsule formulation, whereas all other doses were administered as the F1 capsule formulation. After determination of the recommended phase 2 dose (550 mg/m^2^ of the F1 formulation based on maximum tolerated dose (MTD) from the dose escalation phase), several expansion cohorts were opened in pediatric patients with different solid tumor types and molecular alteration status or in patients requiring entrectinib to be administered by alternative dosing methods. All patients received entrectinib orally with food, once daily in repeated 4-weeks cycles and 2 dose levels (550 mg/m^2^ or 400 mg/m^2^ using F1 capsule) of entrectinib. For patients who could not swallow capsules, the F1 capsules were opened and the contents mixed with liquid or soft food appropriate for the patient’s age and administered orally with food or vial enteral feeding.

The F1 formulation was initially used for all cohorts in the expansion portion, although subsequently the F06 formulation was introduced. Using modeling and simulation approaches rather than MTD, the recommended dose of F06 for patients > 6 months of age was 300 mg/m^2^. Lower doses were recommended for younger pediatric patients (250 mg/m^2^ for 1–6 months, and 100 mg/m^2^ from birth to 1 month) (manuscript in preparation).

An objective of the study was to investigate the pharmacokinetics of single and multiple doses of entrectinib in pediatric patients. Blood samples for entrectinib and M5 plasma concentrations were therefore collected at intervals in both dose escalation and expansion, including intensive sampling on Day 1 of Cycles 1 and 2.

### Patients

The study includes patients from birth to < 18 years (initially young adult patients aged up to 22 years were also allowed). For the expansion phase, patients had one of the following: relapsed or refractory extracranial solid tumors, primary brain tumors with *NTRK1/2/3* or *ROS1* gene fusions or extracranial solid tumors with *NTRK1/2/3* or *ROS1* gene fusions. Patients had measurable or evaluable disease and a life expectancy of at least 4 weeks. Prior cancer therapy was allowed, but had to have been completed within prespecified time limits prior to the start of entrectinib dosing and patients had to have recovered from any acute toxic effects prior to enrollment. Patients receiving enzyme-inducing antiepileptic drugs were excluded. The study was approved by the relevant ethics committees and conducted in accordance with the principles of the Declaration of Helsinki and Good Clinical Practice guidelines.

### Pharmacokinetic assessments

Entrectinib and M5 concentrations were measured using a validated liquid chromatography–tandem mass spectrometry (LC–MS/MS) method with a lower limit of quantification of 2.00 ng/mL. Single dose and steady-state entrectinib and M5 pharmacokinetic parameters were determined from intensive sampling data using noncompartmental analysis (Phoenix WinNonlin software, Certara, NJ, USA). Pharmacokinetic parameters included maximum plasma concentration (C_max_) and area under the curve (AUC) from time zero to the last measurable concentration (AUC_last_).

### Statistical assessments and sample size

The dose escalation portion used a standard “3 + 3” patient enrollment scheme followed by an accelerated titration design, with safety and tolerability being the primary objective, and therefore the sample size was based on the requirement of the study design, of approximately 6 to 30 patients. Sample sizing for the dose expansion portion was not based on pharmacokinetic end points and is not discussed here.

## Results

### Patient disposition and demographics

A total of 16 patients were enrolled into the dose escalation phase. These patients received 250 mg/m^2^ (*n* = 3; F2B), 400 mg/m^2^ (*n* = 3; F1), 550 mg/m^2^ (*n* = 7, F1) and 750 mg/m^2^ (*n* = 3; F1). At the data cutoff (September 2020), a further 27 patients had been enrolled into the dose expansion and received entrectinib as F1 (*n* = 21) or F06 (*n* = 5) or age-appropriate formulation (*n* = 1; data not shown). Patients in the dose expansion received 550 mg/m^2^ F1 (*n* = 12) or 300 mg/m^2^ F06 (*n* = 5), or 400 mg/m^2^ F1 for patients who could not swallow intact capsules (*n* = 9). Of the 43 patients who were enrolled in both parts, 22 were male, 21 were female, and the majority (37) were White. Median age was 7 years (range 2 months to 20 years). A total of 26 patients had tumors with target gene fusions. Of the 43 patients treated, 42 had evaluable pharmacokinetic data.

Twelve patients did not receive the same dose on Day 1 of Cycles 1 and 2 because of dose interruption/dose modification between Cycle 1 and 2. These included six patients treated initially with F1 at 550 mg/m^2^, three patients treated initially with F1 at 750 mg/m^2^, and three patients treated initially with F1 at 400 mg/m^2^.

### Entrectinib pharmacokinetics in pediatric patients (F1 and F2B)

**Fig. 1:**
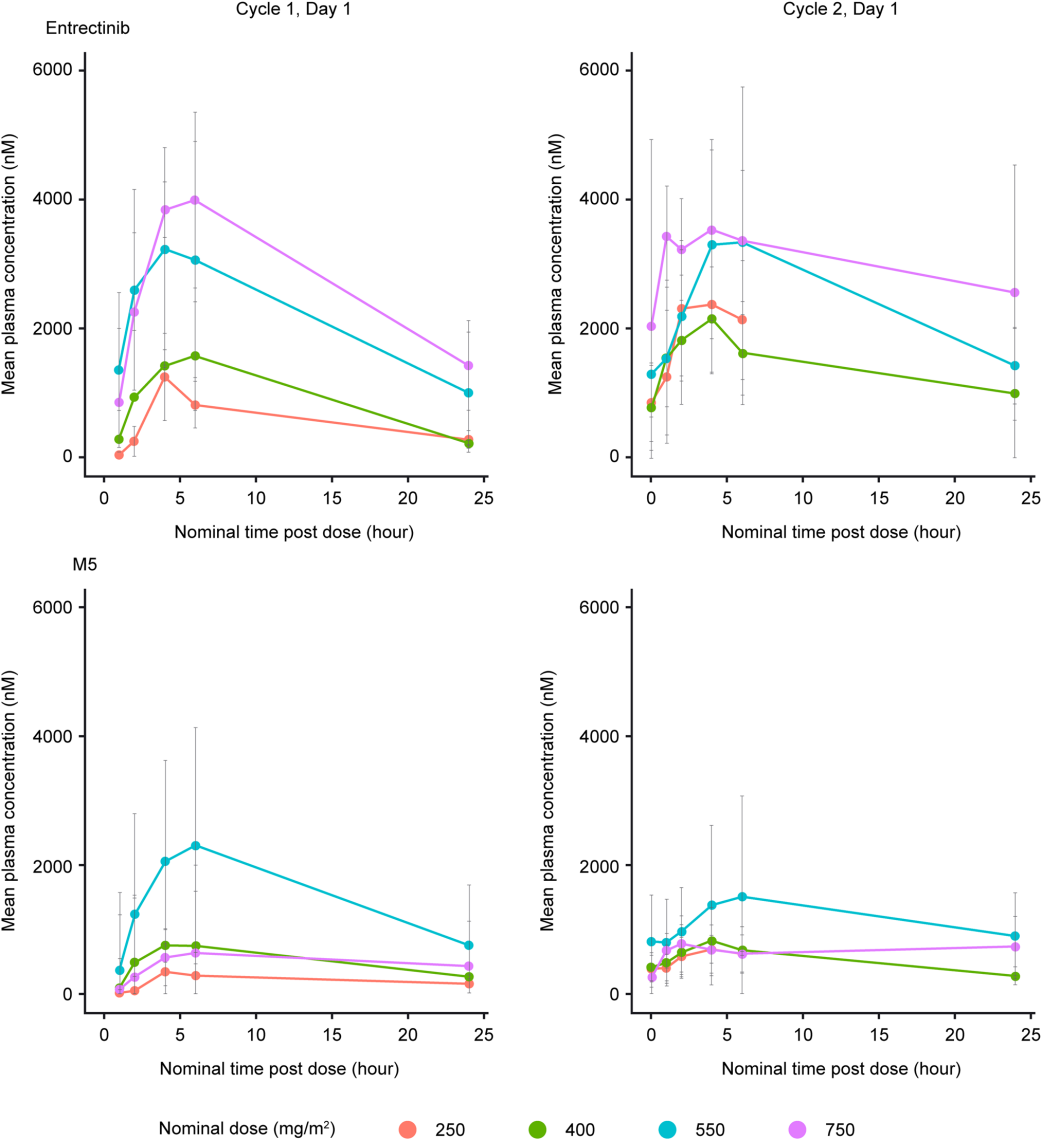
Mean (± SD) entrectinib and M5 plasma concentration–time profiles in pediatric patients with advanced cancers after a single dose (Cycle 1, Day 1) and at steady state (Cycle 2, Day 1) after repeat dosing with entrectinib in the fed state (dose escalation and expansion)

Noncompartmental analysis of the pharmacokinetic data from the 37 patients who received the F1 (*n* = 34) and F2B (*n* = 3) formulations demonstrated that although there was considerable interpatient variability, entrectinib and M5 exposures increased with dose after a single dose of entrectinib across the dose range tested (250–750 mg/m^2^) (Table 1; Fig. 1). Dose-dependent pharmacokinetics following repeat administration of entrectinib was difficult to assess due to the limited number of patients available for pharmacokinetic assessment at steady state (Cycle 2 Day 1), particularly for the 750 mg/m^2^ dose. However, systemic exposure following repeat dosing administration appeared to increase in a supra-proportional manner from 400 mg/m^2^ to 550 mg/m^2^. Data from the 400 mg/m^2^ dose group suggested no apparent differences in the performance of the F1 formulation swallowed intact or opened and the contents mixed with liquid of soft food for administration after single dosing (Table 2). However, caution should be taken when interpreting these data since three out of nine patients in the open capsule group received gastric acid reducing agents covering the Cycle 1 and 2 period. Two individuals in the 550 mg/m^2^ dose group had notably higher exposures after a single dose, for which no obvious cause was identified.

**Table 1:** Summary of entrectinib and M5 pharmacokinetic parameters after single and multiple doses of entrectinib (F1 or F2B formulation) in the fed state in pediatric patients with solid tumors (dose escalation and expansion)

Nominal entrectinib dose	Formulation	Entrectinib		M5	
		C_max_ (μM)	AUC_0-last_ (μM•h)	C_max_ (μM)	AUC_0-last_ (μM•h)
Cycle 1, Day 1					
250 mg/m^2^ (*n* = 3)	F2B	1.13 (71)	12.7 (48)	0.305 (81)	4.17 (75)
400 mg/m^2^ (*n* = 12)	F1	1.71 (34)	22.3 (43)	0.755 (83)	10.4 (79)
550 mg/m^2^ (*n* = 19)	F1	3.20 (55)	41.9 (64)	0.92 (111)	13.8 (108)
750 mg/m^2^ (*n* = 3)	F1	4.19 (26)	62.7 (23.5)	0.653 (16)	11.4 (16)^a^
Cycle 2, Day 1					
250 mg/m^2^ (*n* = 3)	F2B	2.25 (56)	NC	0.626 (67)	NC
400 mg/m^2^ (*n* = 7)	F1	2.01 (41)	25.8 (65)^b^	0.748 (72)	9.69 (72)^b^
550 mg/m^2^ (*n* = 12)	F1	3.48 (37)	58.0 (38)^c^	1.20 (72)	22.2 (66)^c^
750 mg/m^2^ (*n* = 2)	F1	3.81 (42)	NC	0.809 (43)	NC

Values are geometric mean (CV%)

*NC* not calculated because* n* < 2

^a^*N* = 2

^b^*N* = 5

^c^*N* = 7

**Table 2:** Summary of entrectinib pharmacokinetic parameters after a single 400 mg/m^2^ dose either as intact F1 capsules or open F1 capsules with the contents sprinkled on soft food (dose escalation and expansion)

Nominal entrectinib dose	Entrectinib	
	C_max_ (μM)	AUC_0-last_ (μM•h)
Cycle 1, Day 1		
400 mg/m^2^ (*n* = 3) Intact	2.01 (27)	22.4 (33)
400 mg/m^2^ (*n* = 9) Open	1.71 (34)	22.7 (48)

Values are geometric mean (geometric CV%)

### Comparison of pediatric and adult entrectinib pharmacokinetics (F1 and F2A)

**Fig. 2:**
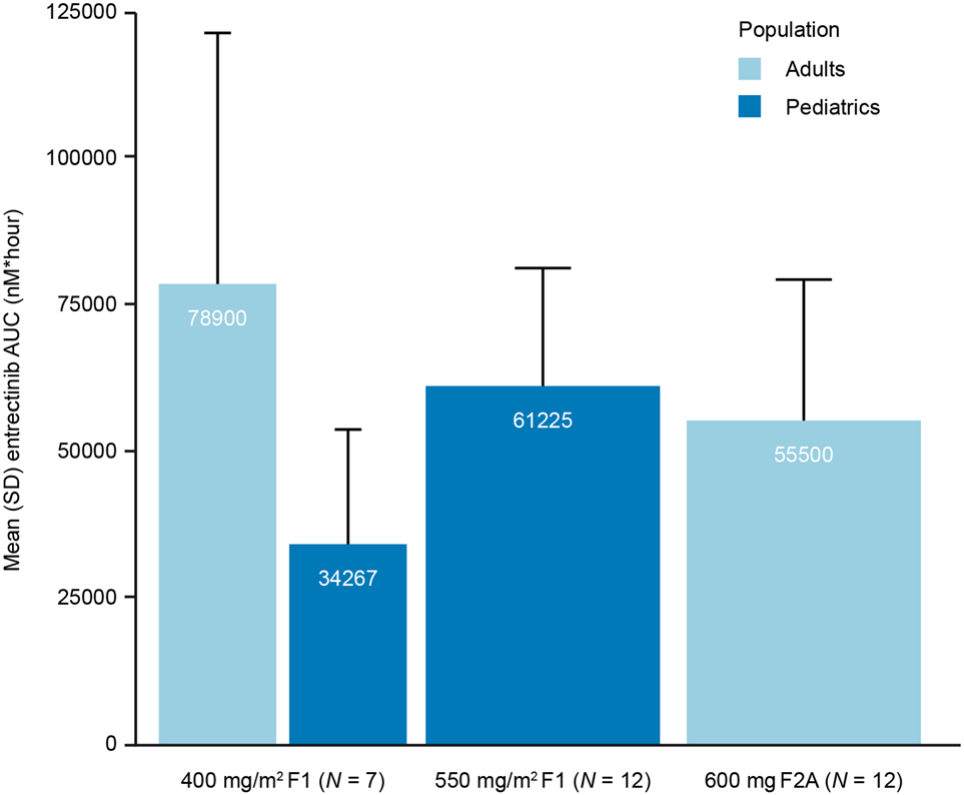
Mean (± SD) entrectinib systemic exposure (AUC_ss_) following multiple doses of 400 mg/m^2^ and 550 mg/m^2^ QD with the F1 formulation in pediatric and adult subjects (Cycle 2, Day 1), or a 600 mg flat dose of the F2A formulation in adult subjects. Data in adults (F1 or F2A formulation) are from STARTRK-1 [7]. AUC, area under curve; SD, standard deviation; QD, once daily

An exploratory comparison of entrectinib systemic exposure at steady state has been conducted between pediatric patients dosed in study STARTRK-NG with either 400 mg/m^2^ (*N* = 7) or 550 mg/m^2^ (*N* = 12) with the F1 formulation, and adult patients dosed with either 400 mg/m^2^ (*N* = 7; F1) or 600 mg flat dose (*N* = 12, F2A) from STARTRK-1 [7]. Mean systemic exposure at steady state (AUC_ss_) in pediatric patients following a dose of either 400 or 550 mg/m^2^ with the non-acidulant formulation F1 was lower than systemic exposure in adults dosed with 400 mg/m^2^ F1 (Fig. 2). The mean AUC_ss_ in pediatric patients following 550 mg/m^2^ (F1) was higher than the AUC_ss_ in adults following 600 mg flat dose (F2A).

### Comparison of research and commercial entrectinib formulations in pediatric patients (F1 and F06)

For this comparison, only the systemic exposure of patients with *NTRK* fusion-positive tumors who showed efficacy following entrectinib administration with the F1 formulation are presented. This allowed reassurance that the systemic exposure achieved with 300 mg/m^2^ with F06 is within the systemic exposure of patients who have shown clinical efficacy.

**Fig. 3:**
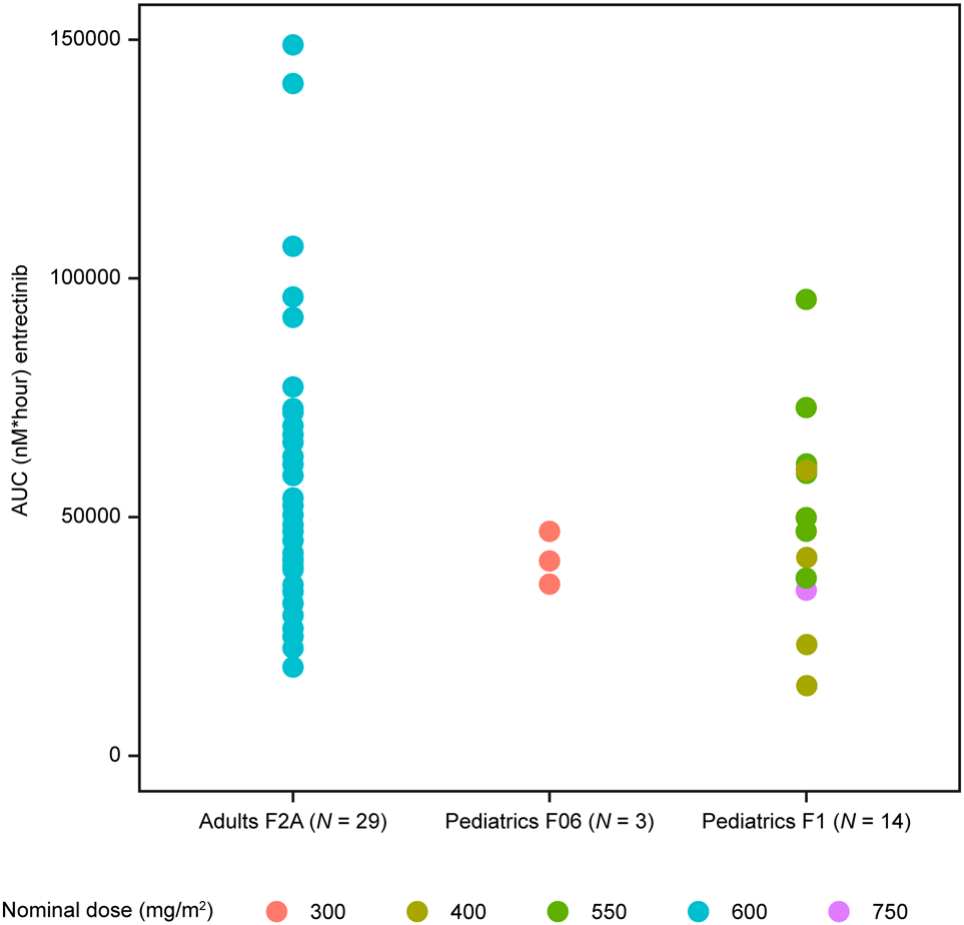
Individual observed entrectinib AUC parameters (Cycle 2, Day 1) for pediatric patients (responders) from STARTRK-NG following 400, 550 or 750 mg/m^2^ QD administration with F1 and following 300 mg/m^2^ administration QD with F06, compared to adults (responders) receiving F2A from STARTRK-2. Three of the five pediatric patients receiving F1 also used gastric acid reducing agents. All adults stopped receiving gastric modifying agents prior to receiving F1. AUC, area under the curve; QD, once daily

At the time of this analysis, five pediatric patients had received the commercial formulation F06 from the start of the treatment. Four patients received the recommended dose of 300 mg/m^2^ and one patient aged 2 months received 250 mg/m^2^ as per protocol. The individual pharmacokinetics data for these patients in shown in Table 3. An exploratory comparison was conducted between C_max_ and AUC_0-last_ estimated using NCA in responder pediatric patients dosed with F1 (*N* = 14) and the pediatric patients dosed with F06 (*N* = 5). Overall, individual systemic exposure in patients receiving 300 mg/m^2^ F06 is within the range of, or higher compared to, the exposure of pediatric patients receiving F1 at doses > 300 mg/m^2^ (i.e., 400, 550, or 750 mg/m^2^) (Fig. 3). In addition, entrectinib systemic exposures in pediatric patients dosed with F06 were within the range of adult responder patients who had tumor responses and received the F2A formulation (bioequivalent to F06).

**Table 3:** Individual observed entrectinib pharmacokinetic parameters for pediatric patients (responders) following 300 mg/m^2^ or 250 mg/m^2^ administration QD with F06 (dose expansion)

Patient ID	Cycle 1, Day 2		Cycle 2, Day 1	
	C_max_ (μM)	AUC_0-last_ (μM•h)	C_max_ (μM)	AUC_0-last_ (μM•h)
A	2.16	26.4	1.96	41.0
B^a^	1.94	25.4	2.89	46.3
C^b^	1.04	11.9		
D	1.76	24.9	2.02	35.9
E	2.60	43.2		

^a^Patient received 550 mg/m^2^ of entrectinib with F1 formulation and switched to F06 on C2D1

^b^Patient received 250 mg/m^2^–recommended dose for children from 1 to 6 months (modeling paper in preparation)

## Discussion

Based on the promising results in adult patients, the unmet medical need, and the rarity of pediatric patients with tumors harboring the *NTRK* gene fusions, the pediatric program was submitted for accelerated approval in various regions in parallel with the adult *NTRK* indication. While substantial efforts were being made to develop an age-appropriate formulation, waiting for the optimal formulation would have significantly delayed access to entrectinib in the pediatric population and therefore clinical development continued with the available formulations of entrectinib capsules. The capsule formulation (F2A) used for adults was only available as a 200 mg dose, whereas the F1 formulation (without acidulant) was available as a 100 mg and 200 mg dose. The majority of the patients in the current study received the F1 formulation due to the availability of the 100 mg capsule needed to dose pediatric patients appropriately. In addition, capsule formulation (F1) could be opened and mixed with food for those pediatric patients unable to swallow capsules. The commercial formulation F06 was developed for both adult and pediatric patients who were able to swallow capsules.

In pediatric patients, although there was considerable interpatient variability, entrectinib and M5 exposures increased with dose after a single dose of entrectinib across the dose range tested (250 to 750 mg/m^2^). The initial recommended phase 2 dose (RP2D) of 550 mg/m^2^ with the clinical F1 formulation was identified based only on safety (MTD) (*N* = 16) using a traditional 3 + 3 MTD study design (Part A) [10]. The decision on the RP2D did not take into account systemic exposures from the dose escalation (Part A) (250 mg/m^2^ to 750 mg/m^2^). The dose recommendation for the commercial F06 formulation was based on a more robust approach by using all the available data, pharmacokinetics and safety in the context of the exposure/efficacy/safety response data available in adults and pediatrics (manuscript in preparation). In order to provide a dose recommendation with the F06 formulation, a population modeling approach built with adult patients treated with the F2A formulation (clinical adult formulation, STARTRK-2) was used to predict the pediatric dose that matches the adult target exposure receiving 600 mg QD of F2A (adult RP2D). Since F2A and F06 have shown bioequivalence, the estimated dose with F2A formulation is the same for F06 formulation and the recommended dose with F06 is 300 mg/m^2^ [7]. The 300 mg/m^2^ dose with F06 triggered questions of potentially underdosing pediatric patients since F1 and F06 have shown to be comparable in healthy adults under controlled dietary conditions helping the performance of F1 (data not shown).

A comparison between the pharmacokinetics in pediatric and adult patients (from STARTRK-1) suggested that pediatric patients receiving 400 mg/m^2^ QD entrectinib using the non-acidulant containing F1 formulation had lower exposures compared to adults receiving the same dose/formulation and also lower exposures than adults receiving the recommended flat dose of 600 mg QD (F2A) (approximately 300 mg/m^2^ for a 70 kg adult). Mean systemic exposure (AUC_ss_) in pediatric patients following a dose of 550 mg/m^2^ F1 was slightly higher than adult systemic exposure when dosed with 600 mg F2A (acidulant containing). It is worth noting that a 600 mg dose in adults corresponds to approximately 300 mg/m^2^; therefore, the systemic exposure in pediatric patients following a dose of 550 mg/m^2^ F1 was expected to be higher than the observed systemic exposure in adults following 600 mg. These results are then consistent with the plausible underperformance of the F1 formulation in the pediatric study described here.

Entrectinib is a basic, lipophilic molecule with significant pH-dependent solubility. The F1 formulation did not contain an acidulant, which made this formulation very sensitive to gastric conditions such as meal size, bile salt solubilization and increased pH caused by comedications [8]. Variability was high in both adult and pediatric patient populations when entrectinib was administered as the F1 formulation [7, 8]. However, the acidulant containing F06 formulation aids dissolution and reduces the sensitivity of entrectinib to type of meals [8]. Due to the smaller gut size in children, the fraction of dose absorbed may be more sensitive to changes in entrectinib solubilization than in adults. This hypothesis is supported by simulations with a mechanistic absorption model developed in GastroPlus [8], which estimated that the reduced bile salt concentrations corresponding to a light meal compared to a high fat meal would result in a drop in simulated fraction absorbed of 37% for a 3-year-old, but only 21% for an adult/young teenager (data not shown). Thus, when the F1 formulation is administered with a lighter meal or sprinkled into just yogurt, the bioavailability in young children may be expected to be more sensitive and variable than in adults. This may partly explain the overall lower systemic exposure in the STARTRK-NG study; however, meal size and composition were not captured in the study. The higher exposure observed in adults compared to pediatric patients following 400 mg/m^2^ with F1 could be attributed to different meal size and composition in the adult population helping the absorption of entrectinib with the non-acidulant F1 formulation [8]. In addition, the request to investigators to avoid the concomitant use of gastric acid reducing agents in the STARTRK-1 adult study could have contributed to the better F1 performance in that study.

When looking at the individual data from study STARTRK-NG, 4 out of 12 pediatric patients dosed with 400 mg/m^2^ received either gastric acid reducing agents (*n* = 1), dexamethasone (*n* = 2) and/or had dose reduction/interruption (*n* = 3). All these factors are expected to contribute to the lower systemic exposure observed in the 400 mg/m^2^ group. In addition, 5 out of 12 pediatric patients treated with 550 mg/m^2^ on Cycle 1, Day 1 had dose reductions/interruptions during Cycle 1, which were ongoing on Cycle 2, Day 1, resulting in lower systemic exposure than would have been expected had they received 550 mg/m^2^ throughout. Furthermore, it is unknown if pediatric patients receiving entrectinib sprinkled onto food ate the entire portion of food. Finally, one pediatric patient received entrectinib via nasogastric administration; however, no formal assessment has been conducted on the impact of entrectinib absorption when using nasogastric administration with F1. This work, together with the modeling approaches described elsewhere (manuscript in preparation), also highlights the need to revisit the traditional MTD approach to select RP2D for molecularly targeted agents. An integrated approach leveraging pediatric, adult systemic exposure and safety together with robust modeling approaches should be implemented early on in the pediatric program to guide dose selection (RP2D). For this to happen, emerging pharmacokinetics data from the pediatric dose escalation study need to be available and integrated in a validated pharmacokinetics model at the time of the RP2D selection.

In summary, the observed pediatric systemic exposures following administration with 300 mg/m^2^ entrectinib with F06 in pediatric patients were comparable to the adult systemic exposure receiving 600 mg QD, and were above the reported IC50s [11]. The presence of the acidulant in the F06 formulation has largely resolved the sensitivity of entrectinib to gastric environment conditions, thus resolving the problem of high variability observed with the F1 formulation. In addition, the emerging efficacy data from all patients who received the recommended dose of F06 have shown overall responses from partial responses to complete responses [10]. Similar efficacy has been demonstrated in adult patients treated with entrectinib [12]. With the recommended dose of 300 mg/m^2^ with F06, or the equivalent based upon the child’s body surface area, children achieve comparable systemic exposure to adults following 600 mg flat dose QD and they thereby reach the target efficacious exposure range while minimizing the risk of overexposure.

## Data Availability

For eligible studies qualified researchers may request access to individual patient-level clinical data through a data request platform. At the time of writing, this request platform is Vivli. https://vivli.org/ourmember/roche/. For up-to-date details on Roche's Global Policy on the Sharing of Clinical Information and how to request access to related clinical study documents, see here: https://go.roche.com/data_sharing. Anonymized records for individual patients across more than one data source external to Roche cannot, and should not, be linked due to a potential increase in risk of patient re-identification.
